# Taxonomic review of Korean *Siphonaria* species (Mollusca, Gastropoda, Siphonariidae)

**DOI:** 10.3897/BDJ.13.e139388

**Published:** 2025-02-18

**Authors:** Yukyung Kim, Jina Park, Ui Wook Hwang, Joong-Ki Park

**Affiliations:** 1 Division of EcoScience, Ewha Womans University, Seoul, Republic of Korea Division of EcoScience, Ewha Womans University Seoul Republic of Korea; 2 Department of Biology Education, Teachers College and Institute for Phylogenomics and Evolution, Kyungpook National University, Daegu, Republic of Korea Department of Biology Education, Teachers College and Institute for Phylogenomics and Evolution, Kyungpook National University Daegu Republic of Korea

**Keywords:** Panpulmonata, Korean *Siphonaria*, taxonomic review, shell morphology, radula, mtDNA *cox1*

## Abstract

**Background:**

Many molluscan species exhibit a high degree of shell morphological plasticity in their shape (including sculptures), size and colour patterns, which can vary significantly depending on environmental conditions. These shell morphological variations make it challenging to differentiate species, based on morphology alone, often resulting in various taxonomic errors, such as misidentifications, overlooking cryptic species diversity or a plethora of nominal species. The genus *Siphonaria* constitutes a significant component of the macrobenthic invertebrate fauna in intertidal habitats across temperate to tropical regions. Given the limited attention to shell variation in previous taxonomic studies on the Korean *Siphonaria* species, the extensive range of ecophenotypic shell variations documented in this group raises questions about the taxonomic validity of previously reported *Siphonaria* species in Korea.

**New information:**

The present study provides a comprehensive taxonomic review of Korean *Siphonaria* species using a combination of shell morphology, radula structure and phylogenetic analysis of the mtDNA *cox1* sequences. This integrative analysis confirmed the validity of *S.acmaeoides*, *S.japonica* and *S.sirius* in Korea, highlighting differences in shell and siphonal groove morphology amongst these species. Detailed descriptions of shell and radula characteristics, along with mtDNA *cox1* sequences as DNA barcodes, are also provided, which are very useful for the accurate identification of *Siphonaria* species. Unlike these three *Siphonaria* species, the taxonomic validity of the four other species (*S.coreensis*, *S.javanica*, *S.laciniosa* and *S.rucuana*) previously reported from Korean waters is questionable, given their documented geographic distribution ranges and the potential misidentification of shell variants in Korean malacofaunal studies.

## Introduction

The genus *Siphonaria* G. B. Sowerby I, 1823, also known as the ‘false limpet’ genus, is a marine panpulmonate group consisting of 106 species worldwide (MolluscaBase eds 2024). *Siphonaria* species constitute a significant component of the macrobenthic community in intertidal/subtidal habitats across temperate to tropical regions, but some are found in cold temperature environments, such as sub-Antarctic shores (Vermeij 1971, Hodgson 1999, Dayrat et al. 2014, González‐Wevar et al. 2018). They are typically inhabit intertidal rocky shores, but are occasionally found at the bottom of rocks or amongst seagrass in the subtidal zone (Collin 2000, Toyohara et al. 2001, Dayrat et al. 2014, Okutani 2000, Okutani 2017). Taxonomically, Sowerby (1823) first introduced the genus name *Siphonaria* with the type species *S.sipho* Sowerby, 1823 (now considered as a junior synonym of *S.javanica* (Lamark, 1819), distinguishing it from the ‘true limpet’ genera *Patella* Linnaeus, 1758 and *Emarginula* Lamarck, 1801 by its posteriorly curved apex and a prominent siphonal groove on the lateral side. The siphonal groove allows animals to perform air-breathing through the mantle cavity, while the secondary gill supports aquatic respiration. These characteristics distinguish them from Patellogastropoda (i.e. true limpets), which also have a similar conical or cap-like shell, but lack the siphonal groove, relying solely on gills for their respiration.

*Siphonaria* species exhibit remarkedly high ecophenotypic shell variation, leading to many taxonomic complications such as species misidentification and the potential for unrecognised cryptic species (White and Dayrat 2012, Dayrat et al. 2014, De Coito 2021). To date, seven species have been recorded in Korea (National Institute of Biological Resources 2023, Jenkins and Köhler 2024): *S.acmaeoides* Pilsbry, 1894, *S.coreensis* A. Adams and Reeve, 1848, *S.japonica* (Donovan, 1824), *S.javanica* (Lamarck, 1819), *S.laciniosa* (Linnaeus, 1758), *S.sirius* Pilsbry 1894, and *S.rucuana* Pilsbry, 1904. Of these, *S.coreensis* is designated as a "*taxon inquirendum*" in the World Register of Marine Species (MolluscaBase eds 2025), reflecting the view of several authorities who have suggested that it may represent a misidentification of *S.atra* Quoy & Gaimard, 1833 (Lischke 1871, Shikama 1964, Cernohorsky 1972, Jenkins and Köhler 2024). Furthermore, its occurrence in Korea, as well as northern Pacific Region, has not been substantiated since its initial report. In addition, most previous Korean *Siphonaria* records relied primarily on species checklists or taxonomic illustrations, lacking detailed descriptions of diagnostic characters and overlooking individual shell variation in their species identification. Given the limited attention to shell variation in previous studies, the extensive ecophenotypic morphological plasticity observed in this group raises questions regarding the taxonomic validity of previously reported Korean *Siphonaria* species. Insufficient and/or erroneous taxonomic information has continued to pose challenges not only in taxonomy, but also in other scientific fields that rely on accurate species identification.

In this study, we re-examined the taxonomic validity of six *Siphonaria* species previously reported in Korea by conducting a comprehensive comparison of morphological characters described in previous records against the original descriptions, based on an in-depth analysis of shell and radula morphologies, combined with mtDNA *cox1* sequence data. Based on the results of this taxonomic re-assessment, we provide detailed morphological descriptions of the shell morphology and radula microstructures of three *Siphonaria* species (*S.acmaeoides*, *S.japonica* and *S.sirius*) confirmed to occur along the Korean coast using scanning electron microscopy (SEM), as well as a phylogenetic analysis of the mtDNA *cox1* sequences.

## Materials and methods

**Figure 1.**
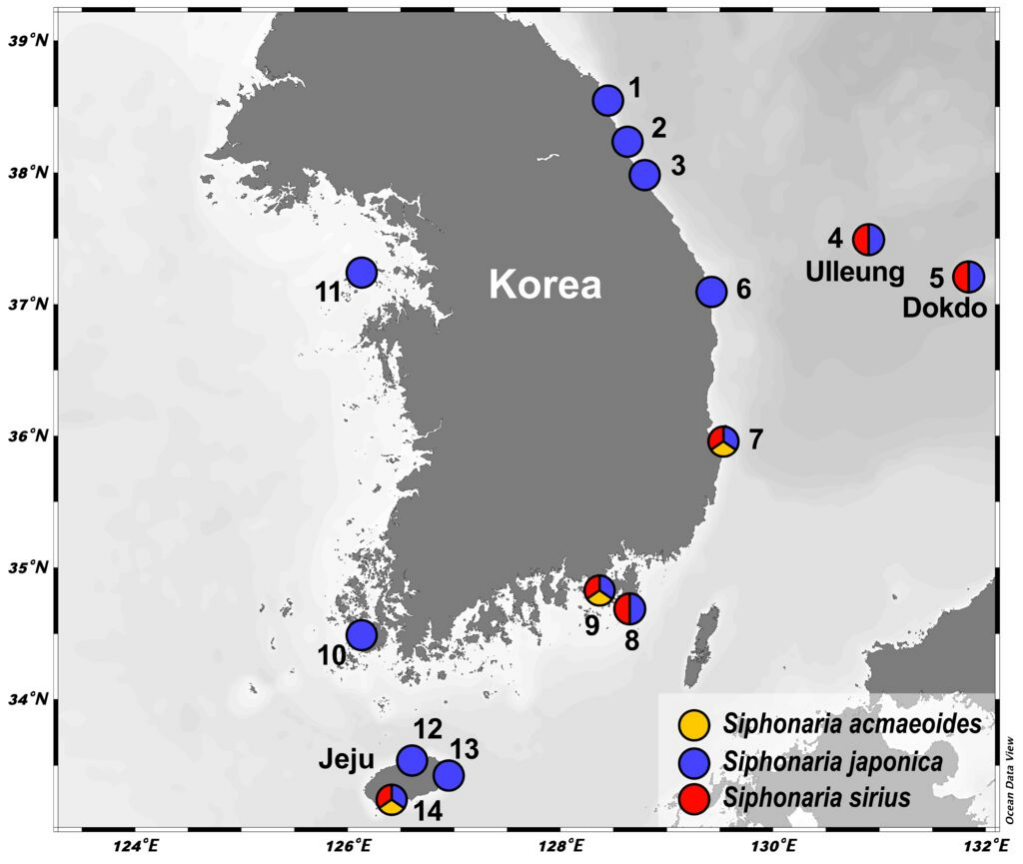
Map showing the sampling localities for Korean *Siphonaria* examined in this study. **1** Daejin-ri, Hyeonnae-myeon, Goseong-gun, Gangwon-do; **2** Jeonjin-ri, Ganghyeon-myeon, Yangyang-gun, Gangwon-do; **3** Namae-ri, Hyeonnam-myeon, Yangyang-gun, Gangwon-do; **4** Namyang-ri, Seo-myeon, Ulleung-gun, Gyeongsangbuk-do; **5** Dokdo-ri, Ulleung-eup, Ulleung-gun, Gyeongsangbuk-do; **6** Jukbyeon-ri, Jukbyeon-myeon, Uljin-gun, Gyeongsangbuk-do; **7** Guryongpo-eup, Nam-gu, Pohang-si, Gyeongsangbuk-do; **8** Gujora-ri, Irun-myeo, Geoje-si, Gyeongsangnam-do; **9** Mendehaean-gil, Tongyeong-si, Gyeongsangnam-do; **10** Gahak-ri, Jisan-myeon, Jindo-gun, Jeollanam-do; **11** Seopo-ri, Deokjeok-myeon, Ongjin-gun, Incheon; **12** Jeju-si Samyang 1(il)-dong, Jeju-do; **13** Seongsan-eup, Seogwipo-si, Jeju-do; **14** Andeok-myeon, Seogwipo-si, Jeju-do. Circles with multiple colours represent localities where the corresponding species were found together.

Specimens were collected from intertidal rocky shores in Korea (Fig. 1) and preserved in 95% ethanol solution. For species identification and morphological descriptions, shells were observed using a stereoscopic microscope (Leica M205C, Wetzlar, Germany) and radula characters were examined using a scanning electron microscope (SEM). To prepare the radula for SEM examination, the radula sac was extracted from the buccal mass and residual tissue was dissolved in a mixture of 180 μl ATL buffer and 20 μl Proteinase K at 56°C for about 1 hour. Then the radula was rinsed with distilled water and ethanol, mounted on nickel tape attached to an SEM stub, air-dried and coated with platinum. Radula images were captured using an SEM (Zeiss Ultra Plus, Germany) and a FE-SEM (Jeol JSM-7800F, Japan). The examined specimens are deposited at the National Institute of Biological Resources (NIBR) in Incheon, Korea and the Animal Phylogenomics Laboratory at Ewha Womans University, Seoul, Korea.

For sequencing of the mtDNA *cox1* gene fragments, genomic DNA was extracted from foot tissue by using a DNeasy Blood and Tissue kit (QIAGEN, Germany) following the manufacturer’s protocol. The partial mitochondrial *cox1* gene sequence was amplified by polymerase chain reaction (PCR) using the primer pair LCO1490 and HCO2198 (Folmer et al. 1994). Amplifications were performed in a 50 μl total volume, containing 3 μl of template DNA, 35.75 μl of distilled water, 5 μl of 10x Ex Taq buffer, 1 μl of each primer, 4 μl of dNTP and 0.25 μl of TaKaRa Ex Taq DNA polymerase (TaKaRa Bio, Japan) under the following conditions: an initial denaturation at 95℃ for 5 min, 40 cycles of denaturation at 94℃ for 40 s, annealing at 48℃ for 1 min, elongation at 72℃ for 1 min and a final extension at 72℃ for 10 min. The PCR-amplified target fragment was purified using a Qiaquick gel extraction kit (Qiagen Valencia, USA), sequenced using an ABI PRISM 3730xl DNA analyser (Applied Biosystems, USA) and analysed using Geneious Prime v. 2022.2.2 (Biomaters, Auckland, New Zealand).

The newly-determined *cox1* sequences of *S.acmaeoides*, *S.japonica* and *S.sirius* were deposited in GenBank (accession numbers in Table 1). The *cox1* sequences from the three Korean *Siphonaria* species and the homologous gene sequences from NCBI database (Table 1) were used for phylogenetic analysis with two outgroup species; *Trimusculusafer* (Gmelin, 1791) and *T.reticulatus* (G. B. Sowerby I, 1835). The sequences were aligned using the default settings of MUSCLE (Edgar 2004) in the Geneious software and trimmed to a length of 630 bp. MEGA X was used to calculate genetic distances within and between species, applying uncorrected *p*-distance (Collins et al. 2012; Srivathsan and Meier 2012). To select the best-fit nucleotide substitution model, ModelFinder (Kalyaanamoorthy et al. 2017), implemented in IQ-TREE v.1.6.12. Nguyen et al. (2015) was applied with the corrected Akaike Information Criterion (AICc). Phylogenetic analysis was performed using Maximum Likelihood (ML) estimation with IQ-TREE software, with the K3Pu+F+I+G4 substitution model and 1,000 standard bootstrap pseudoreplicates (Felsenstein 1985) to assess branch support values.

## Taxon treatments

### Siphonaria acmaeoides

Pilsbry, 1894

7AFBE1FC-ED80-5A0E-BA7D-0D7D215CF3A2

https://www.gbif.org/species/6787904

https://www.marinespecies.org/aphia.php?p=taxdetails&id=599431

Siphonaria acmaeoides Pilsbry, 1894: *Pilsbry 1894*: 16; Pilsbry 1895: 6, pl. 6, figs. 19–22; Lee 1956: 79; Hirase 1941: 94, pl. 121, fig. 15; Kuroda and Habe 1952: 86 (cited from Jenkins and Köhler 2024); Azuma 1960: 62 (cited from Jenkins and Köhler (2024)); Baker 1964: 159 (cited from Jenkins and Köhler (2024)); Christiaens 1980b: 466; White and Dayrat 2012: 60 [checklist]; Jenkins and Köhler 2024: 114, 115, figs. 43A–D, M, N, 44A–C.
Siphonaria (Patellopsis) acmaeoides : Hubendick 1945: 70, fig. 19 (cited from Jenkins and Köhler (2024)); Hubendick 1946: 30, pl. 6, figs. 12–15; Habe and Kikuchi 1960: 64 (cited from Jenkins and Köhler (2024)); Kira 1962: 201, pl. 69, fig. 9a, b (misspelled as '*Patellops*'; cited from Jenkins and Köhler (2024)).
Siphonaria zebra : Kuroda and Habe 1952: 86 (non *Siphonariazebra* Reeve, 1856) (cited from Jenkins and Köhler (2024)).
Planesiphon acmaeoides : Kuroda et al. 1971: 484, pl. 64, fig. 8; Choe 1992: 749, pl. 126, fig. 224.
Siphonaria (Mouretus) acmaeoides : Christiaens 1980a: 79, 80.
Siphonaria (Planesiphon) acmaeoides : Inaba 1983: 149 (cited from Jenkins and Köhler (2024)); Je 1989: 29 [checklist]; Higo et al. 1999: 402, G4976 [checklist]; Okutani 2000: 815, pl. 405, fig. 7; Cheng et al. 2005: 8, fig. 1e–g; Noseworthy et al. 2007: 90 [checklist]; Okutani 2017: 1101, pl. 403, fig. 7.

#### Materials

- **Type status:** Other material. **Occurrence:** catalogNumber: NIBRIV0000307707; individualCount: 1; occurrenceID: 83FCD050-F70C-5128-B42F-DE6C1A9BA020; **Taxon:** scientificName: *Siphonariaacmaeoides*; kingdom: Animalia; phylum: Mollusca; class: Gastropoda; order: Siphonariida; family: Siphonariidae; genus: Siphonaria; specificEpithet: *acmaeoides*; scientificNameAuthorship: Pilsbry, 1894; **Location:** country: Korea; locality: Hyeongjehaean-ro, Andeok-myeon, Seogwipo-si, Jeju-do; verbatimCoordinates: 33°13'37.13"N 126°18'30.92"E; **Event:** eventDate: 2012-01-07
- **Type status:** Other material. **Occurrence:** individualCount: 3; occurrenceID: D7664B8A-483D-5DD2-9933-CECE40648E65; **Taxon:** scientificName: *Siphonariaacmaeoides*; kingdom: Animalia; phylum: Mollusca; class: Gastropoda; order: Siphonariida; family: Siphonariidae; genus: Siphonaria; specificEpithet: *acmaeoides*; scientificNameAuthorship: Pilsbry, 1894; **Location:** country: Korea; locality: Sagye-ro, Andeok-myeon, Seogwipo-si, Jeju-do; verbatimCoordinates: 33°13'13.2"N 126°17'42.0"E; **Event:** eventDate: 2022-04-21
- **Type status:** Other material. **Occurrence:** individualCount: 3; occurrenceID: A1D5EA8B-4851-5AD4-A0A1-2FCC3E1B0328; **Taxon:** scientificName: *Siphonariaacmaeoides*; kingdom: Animalia; phylum: Mollusca; class: Gastropoda; order: Siphonariida; family: Siphonariidae; genus: Siphonaria; specificEpithet: *acmaeoides*; scientificNameAuthorship: Pilsbry, 1894; **Location:** country: Korea; locality: Donghaean-ro, Guryongpo-eup, Nam-gu, Pohang-si, Gyeongsangbuk-do; verbatimCoordinates: 35°57'05.7"N 129°33'04.4"E; **Event:** eventDate: 2023-07-23
- **Type status:** Other material. **Occurrence:** individualCount: 1; occurrenceID: 84F9DDD5-5BD5-5645-8D86-F3D843A9ACD2; **Taxon:** scientificName: *Siphonariaacmaeoides*; kingdom: Animalia; phylum: Mollusca; class: Gastropoda; order: Siphonariida; family: Siphonariidae; genus: Siphonaria; specificEpithet: *acmaeoides*; scientificNameAuthorship: Pilsbry, 1894; **Location:** country: Korea; locality: Irun-myeon, Geoje-si, Gyeongsangnam-do; verbatimCoordinates: 34°58'34.9"N 128°41'18.18"E; **Event:** eventDate: 2023-04-19

#### Description

Measurements: Shell length [SL] 11.32–17.58 mm, Shell width [SW] 9.42–14.87 mm, Shell height [SH] 2.63–6.73 mm.

Shell (Fig. 2a, b) solid, oval, small to medium in size (in examined specimens, SL 11.32–17.58 mm), medium-high in height (about 2/7 on its length). Exterior colour generally greyish-brown or yellowish-brown with dark brown irregular maculations. Shell surface with 14–17 wide, light-cream or white-coloured primary ribs, unevenly spaced, sometimes discontinuous from apex to outer shell margin in juvenile. Interspaces between primary ribs filled with 1–7 thin secondary ribs. Apex white, glossy, spiral in counterclockwise direction (Fig. 2bC), often eroded due to shell growth, positioned in posterior one-third of shell, biased towards left. Shell margin weakly undulated. Anterior slope slightly convex, posterior slope short, nearly straight. Siphonal groove indistinct on outer shell surface. Interior colour dark brown, reddish-brown, with light cream bands near margin, reddish-brown or pale at centre.

Radula (Fig. 3a, b) dentition formulae 27:1:27 (in specimens measuring SL 16.23 mm, SW 11.88 mm, SH 4.80 mm). Each transverse row with narrow central rachidian tooth flanked by symmetrical half rows with lateral teeth, decreasing in size outwards. Rachidian tooth (Fig. 3a) short, approximately one-third length of lateral tooth, with sharply-pointed cusp. Innermost lateral teeth (first 7 lateral teeth), each tooth consisting of mesocone without ectocone; mesocone with bicuspid (Fig. 3a). Middle lateral teeth (following 5 lateral teeth), each tooth consisting of mesocone and ectocone; mesocone with bicuspid to blunt towards the outer margins; ectocone sharply pointed. Outermost lateral teeth (remaining 12 lateral teeth) consisting of endocone, ectocone and mesocone; mesocone subquadrate-shaped; endocone and ectocone short, sharply pointed (Fig. 3b).

#### Distribution

Korea, Japan and Taiwan.

**Type locality**: Japan; Boshiu island (Boso Peninsula).

**Habitat**: On rocky substrate in high intertidal zones, typically in shallow pools (Fig. 2aA).

#### Taxon discussion

**Figure 4.**
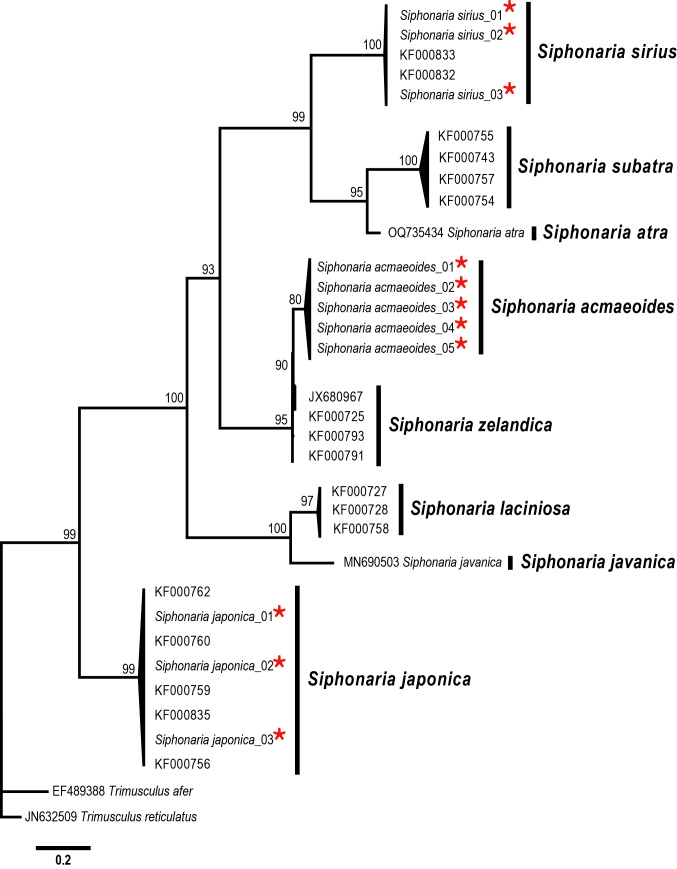
Phylogenetic relationships amongst some selected NWP *Siphonaria* species inferred from the Maximum Likelihood method using the mtDNA *cox1* sequences. The bootstrap supporting values (≥ 50%) are indicated on the branches. Asterisks (*) represents the mtDNA *cox 1* sequences determined in this study.

The Korean *S.acmaeoides* corresponds well with Pilsbry (1894)'s original description. The external shell morphology is characterised by wide, low primary ribs with fine secondary ribs in the interspaces, a glossy spiral apex coiling counterclockwise and a weakly undulated shell margin. Christiaens (1980a) described a new subspecies, *S.acmaeoidespaulae* Christiaens, 1980, from Hong Kong, distinguishing it by its finer, thinner, more elliptical, lighter-coloured shell. This subspecies was also noted to lack a marked central area and exhibit a more bulging siphon compared to *S.acmaeoides* reported from Japan. However, Jenkins and Köhler (2024) later synonymised *S.a.paulae* with the *S.acmaeoides*, citing the morphological similarity of the shell. Based on our examination of the Korean specimens (Fig. 2b), we did not observe the light-coloured shell characteristics described in *S.a.paulae* by Christiaens (1980a). Further morphological and molecular analyses for *S.a.paulae* sampled from Hong Kong are necessary to confirm the taxonomic validity of this subspecies. In addition, *S.acmaeoides* has repeatedly been noted for its similarity in shell and radula morphology to *S.zelandica* Quoy and Gaimard, 1833 which is distributed in Australia (Hubendick 1946, Dayrat et al. 2014, Jenkins and Köhler 2024). Both species share the following morphological characteristics: a homostrophic apex, broad primary ribs with fine secondary ribs and an innermost lateral tooth in the radula with a mesocone that lacks ectocone and endocone (Quoy and Gaimard 1833, Pilsbry 1894, Jenkins 1983, Jenkins and Köhler 2024). Hubendick (1946) reported the radula formula of *S.acmaeoides* as 26:1:26, noting the absence of an ectocone on the lateral teeth. In this study, we observed a similar pattern of radula characters, with a radula formula of 27:1:27, also lacking an ectocone on the lateral teeth. This can be generally included within the documented range of intraspecific variation for the genus *Siphonaria* (e.g. in *S.lessonii* Blaninville, 1827, the radula formula varies from 13:1:13 to 76:1:76; Hubendick (1946), Güller et al. (2016)). In shell morphology, *S.acmaeoides* is distinguished by having its less prominent, almost flat radial ribs and a more indistinct siphonal groove, whereas *S.zelandica* is characterised by relatively finer and dual radial ribs within the siphonal groove. In our phylogenetic tree, the two species form a sister clade with 95% bootstrap support (Fig. 4). The uncorrected *p*-distances between *S.acmaeoides* and *S.zelandica* range from 5.71 to 6.51%, while *S.sirius*, the next closest related species shows a significantly higher genetic divergence (27.94%) (Table 2). Thus, a taxonomic re-assessment of the two species is necessary to confirm their taxonomic validity.

### Siphonaria japonica

(Donovan, 1824)

41D3904D-99D3-5888-A457-AFB80F10DAF6

https://www.gbif.org/species/5859486

https://www.marinespecies.org/aphia.php?p=taxdetails&id=740941

Patella japonica Donovan 1824: pl. 79; Kuroda et al. 1971: 483, pl. 64, fig. 7; White and Dayrat 2012: 64 [checklist].
? *Siphonariaradiata*: Adams and Reeve 1850: 69, pl. 13, fig. 2.
? *Siphonriaradians*: *Adams and Adams 1855*: 271; Hubendick 1946: 43.
Siphonaria cochleariformis : *Reeve 1856*: pl. 6, fig. 28; Hubendick 1946: 43, pl. 2, figs. 33–35; Kuroda et al. 1971: 483, pl. 64, fig. 7.
Siphonaria japonica : Hanley 1858: 152 [checklist]; Pilsbry 1920: 141; Abe 1940: 59 (cited from Jenkins and Köhler (2024)); Hirase 1941: 94, pl. 121, fig. 12; Kuroda 1941: 137; Hubendick 1945: 27, fig. 34, 36, 39, 41 (cited from Jenkins and Köhler (2024)); Kuroda and Habe 1952: 86 (cited from Jenkins and Köhler (2024)); Lee 1956: 79; Kuroda 1960: 43 (cited from Jenkins and Köhler (2024)); Habe and Kikuchi 1960: 64 (cited from Jenkins and Köhler 2024); Shikama 1964: 6 (cited from Jenkins and Köhler (2024)); Berry 1977: 197, fig. 19 (cited from Jenkins and Köhler (2024)); Lee and Chao 2003: 35, pl. 6, fig. 173; Qi 2004: 198, pl. 108, fig. E; Dayrat et al. 2014: 266, fig. 3B; Jenkins and Köhler 2024: 31–34, figs. 13A–L, Q–S, 15B–E.
Siphonaria alterniplicata : Grabau and King 1928: 237, pl. 11, fig. 117; Hubendick 1946: 62; White and Dayrat 2012: 60 [checklist]; Coan et al. 2015: 221, fig. 38A, B.
Siphonaria (Sacculosiphonaria) japonica : *Hubendick 1946*: 42, pl. 2, figs. 29–32; Kira 1962: 201, pl. 69, fig. 8a, b (cited from Jenkins and Köhler (2024)); Habe 1971: 15, pl. 4, fig. 16 (cited from Jenkins and Köhler (2024)); Yoo 1976: 89, pl. 19, figs. 1–4; Christiaens 1980a: 79; Christiaens 1980b: 466, 467; Je 1989: 89 [checklist]; Fukuda et al. 1992: 76, pl. 23, fig. 360a, b (cited from Jenkins and Köhler (2024)); Kwon et al. 1993: 334, fig. 61-1; Okutani 2000: 815, pl. 405, fig. 6; Kwon 2001: 191, fig. 723; Hamamura 2004: 115; Min et al. 2004: 335, fig. 1056; Noseworthy et al. 2007: 90 [checklist]; Okutani 2017: 1101, pl. 403, fig. 6.
Sacculosiphonaria japonica : Kuroda et al. 1971: 483, pl. 64, fig. 7; Trew 1983: 9 (cited from Jenkins and Köhler (2024)); Choe 1992: 747, 748, pl. 125, fig. 222.
Siphonaria (Mastosiphon) sirius : Yoo 1976: 89, pl. 19, fig. 5 (non *Siphonariasirius* Pilsbry 1894).
Siphonaria acmaeoides : Kwon et al. 1993: 335, fig. 61-3 (non-*Siphonariaacmaeoides* Pilsbry 1894).
Siphonaria (Planesiphon) acmaeoides : Kwon 2001: 191, fig. 724; Min et al. 2004: 337, fig. 1057 (non-*Siphonariaacmaeoides* in Pilsbry (1894)).
Siphonaria japonica tall form: Yokogawa et al. 2010: fig. 1A.

#### Materials

- **Type status:** Other material. **Occurrence:** catalogNumber: NIBRIV0000894428–NIBRIV0000894447; individualCount: 20; occurrenceID: 8CB2FFA8-7177-5D58-9987-D944894409D8; **Taxon:** scientificName: *Siphonariajaponica*; kingdom: Animalia; phylum: Mollusca; class: Gastropoda; order: Siphonariida; family: Siphonariidae; genus: Siphonaria; specificEpithet: *japonica*; scientificNameAuthorship: (Donovan, 1824); **Location:** country: Korea; locality: Namyang-ri, Seo-myeon, Ulleung-gun, Gyeongsangbuk-do; verbatimCoordinates: 37°27'34.5"N, 130°51'27.4"E; **Event:** eventDate: 2011-11-13
- **Type status:** Other material. **Occurrence:** catalogNumber: NIBRIV0000894458–NIBRIV0000894467; individualCount: 10; occurrenceID: E2FA26B9-DF79-5C3B-BAA0-2A7982854C38; **Taxon:** scientificName: *Siphonariajaponica*; kingdom: Animalia; phylum: Mollusca; class: Gastropoda; order: Siphonariida; family: Siphonariidae; genus: Siphonaria; specificEpithet: *japonica*; scientificNameAuthorship: (Donovan, 1824); **Location:** country: Korea; locality: Jeonjin-ri, Ganghyeon-myeon, Yangyang-gun, Gangwon-do; verbatimCoordinates: 38°07'31.5"N, 128°37'52.3"E; **Event:** eventDate: 2014-04-09
- **Type status:** Other material. **Occurrence:** catalogNumber: NIBRIV0000894468–NIBRIV0000894487; individualCount: 20; occurrenceID: 59D7504B-74E8-514D-BCCA-3C6F3557C300; **Taxon:** scientificName: *Siphonariajaponica*; kingdom: Animalia; phylum: Mollusca; class: Gastropoda; order: Siphonariida; family: Siphonariidae; genus: Siphonaria; specificEpithet: *japonica*; scientificNameAuthorship: (Donovan, 1824); **Location:** country: Korea; locality: Jukbyeon-ri, Jukbyeon-myeon, Uljin-gun, Gyeongsangbuk-do; verbatimCoordinates: 37°3'32.50"N, 129°25'43.92"E; **Event:** eventDate: 2014-04-09
- **Type status:** Other material. **Occurrence:** catalogNumber: NIBRIV0000894448NIBRIV0000894450; individualCount: 3; occurrenceID: 8451E1F2-DB21-501A-A3C9-52209A958584; **Taxon:** scientificName: *Siphonariajaponica*; kingdom: Animalia; phylum: Mollusca; class: Gastropoda; order: Siphonariida; family: Siphonariidae; genus: Siphonaria; specificEpithet: *japonica*; scientificNameAuthorship: (Donovan, 1824); **Location:** country: Korea; locality: Namae-ri, Hyeonnam-myeon, Yangyang-gun, Gangwon-do; verbatimCoordinates: 37°56'33.7"N, 128°47'17.8"E; **Event:** eventDate: 2014-08-24
- **Type status:** Other material. **Occurrence:** catalogNumber: NIBRIV0000894548-NIBRIV0000894562; individualCount: 15; occurrenceID: 7E7E0FC6-5FF9-5A57-A3BD-6F006D828021; **Taxon:** scientificName: *Siphonariajaponica*; kingdom: Animalia; phylum: Mollusca; class: Gastropoda; order: Siphonariida; family: Siphonariidae; genus: Siphonaria; specificEpithet: *japonica*; scientificNameAuthorship: (Donovan, 1824); **Location:** country: Korea; locality: Samyang 1(il)-dong Jeju-si Jeju-do; verbatimCoordinates: 33°31'34.6"N, 126°35'09.0"E; **Event:** eventDate: 2015-12-21
- **Type status:** Other material. **Occurrence:** catalogNumber: NIBRIV0000894451-NIBRIV0000894457; individualCount: 7; occurrenceID: CC219B8C-1E0B-5581-B327-D492B0178063; **Taxon:** scientificName: *Siphonariajaponica*; kingdom: Animalia; phylum: Mollusca; class: Gastropoda; order: Siphonariida; family: Siphonariidae; genus: Siphonaria; specificEpithet: *japonica*; scientificNameAuthorship: (Donovan, 1824); **Location:** country: Korea; locality: Namae-ri, Hyeonnam-myeon, Yangyang-gun, Gangwon-do; verbatimCoordinates: 37°56'33.7"N, 128°47'17.8"E; **Event:** eventDate: 2016-03-02
- **Type status:** Other material. **Occurrence:** catalogNumber: NIBRIV0000894408-NIBRIV0000894424; individualCount: 17; occurrenceID: 2CD256A6-3489-5322-9FA4-8F75AF0BCF39; **Taxon:** scientificName: *Siphonariajaponica*; kingdom: Animalia; phylum: Mollusca; class: Gastropoda; order: Siphonariida; family: Siphonariidae; genus: Siphonaria; specificEpithet: *japonica*; scientificNameAuthorship: (Donovan, 1824); **Location:** country: Korea; locality: Dokdo-ri, Ulleung-eup, Ulleung-gun, Gyeongsangbuk-do; verbatimCoordinates: 37°14'20.12"N, 131°52'05.74"E; **Event:** eventDate: 2016-06-22
- **Type status:** Other material. **Occurrence:** catalogNumber: NIBRIV0000894508-NIBRIV0000894527; individualCount: 20; occurrenceID: 0D774AF9-C5DA-5C87-B0AE-26873ED2A1F7; **Taxon:** scientificName: *Siphonariajaponica*; kingdom: Animalia; phylum: Mollusca; class: Gastropoda; order: Siphonariida; family: Siphonariidae; genus: Siphonaria; specificEpithet: *japonica*; scientificNameAuthorship: (Donovan, 1824); **Location:** country: Korea; locality: Gahak-ri, Jisan-myeon, Jindo-gun, Jeollanam-do; verbatimCoordinates: 34°25'48.1"N, 126°05'55.6"E; **Event:** eventDate: 2016-07-08
- **Type status:** Other material. **Occurrence:** catalogNumber: NIBRIV0000894563-NIBRIV0000894564; individualCount: 2; occurrenceID: EE49E3D2-2BA0-5D88-8772-0303B4B71649; **Taxon:** scientificName: *Siphonariajaponica*; kingdom: Animalia; phylum: Mollusca; class: Gastropoda; order: Siphonariida; family: Siphonariidae; genus: Siphonaria; specificEpithet: *japonica*; scientificNameAuthorship: (Donovan, 1824); **Location:** country: Korea; locality: Ojo-ri, Seongsan-eup, Seogwipo-si, Jeju-do; verbatimCoordinates: 33°28'14.8"N, 126°55'21.9"E; **Event:** eventDate: 2017-06-27
- **Type status:** Other material. **Occurrence:** catalogNumber: NIBRIV0000894565-NIBRIV0000894567; individualCount: 3; occurrenceID: 0CBFE4F7-A55D-52DF-AAC8-16500149F3AD; **Taxon:** scientificName: *Siphonariajaponica*; kingdom: Animalia; phylum: Mollusca; class: Gastropoda; order: Siphonariida; family: Siphonariidae; genus: Siphonaria; specificEpithet: *japonica*; scientificNameAuthorship: (Donovan, 1824); **Location:** country: Korea; locality: Hwasun-ri, Andeok-myeon, Seogwipo-si, Jeju-do; verbatimCoordinates: 33°14'20.7"N, 126°20'01.6"E; **Event:** eventDate: 2017-06-28
- **Type status:** Other material. **Occurrence:** catalogNumber: NIBRIV0000894488-NIBRIV0000894507; individualCount: 20; occurrenceID: BEF4F2B8-098D-5566-8920-C4545FAEE777; **Taxon:** scientificName: *Siphonariajaponica*; kingdom: Animalia; phylum: Mollusca; class: Gastropoda; order: Siphonariida; family: Siphonariidae; genus: Siphonaria; specificEpithet: *japonica*; scientificNameAuthorship: (Donovan, 1824); **Location:** country: Korea; locality: Seopo-ri, Deokjeok-myeon, Ongjin-gun, Incheon; verbatimCoordinates: 37°13'03.16"N, 126°06'55.67"E; **Event:** eventDate: 2018-04-17
- **Type status:** Other material. **Occurrence:** catalogNumber: NIBRIV0000894528-NIBRIV0000894547; individualCount: 20; occurrenceID: B872D531-A994-59F3-A5E9-E1FDD9EAB14F; **Taxon:** scientificName: *Siphonariajaponica*; kingdom: Animalia; phylum: Mollusca; class: Gastropoda; order: Siphonariida; family: Siphonariidae; genus: Siphonaria; specificEpithet: *japonica*; scientificNameAuthorship: (Donovan, 1824); **Location:** country: Korea; locality: Gujora-ri, Irun-myeon, Geoje-si, Gyeongsangnam-do; verbatimCoordinates: 34°48'31.93"N, 128°41'25.97"E; **Event:** eventDate: 2018-06-12
- **Type status:** Other material. **Occurrence:** catalogNumber: NIBRIV0000894425-NIBRIV0000894427; individualCount: 3; occurrenceID: 3B25F340-A40E-5225-BBC0-AC7D16CA5F33; **Taxon:** scientificName: *Siphonariajaponica*; kingdom: Animalia; phylum: Mollusca; class: Gastropoda; order: Siphonariida; family: Siphonariidae; genus: Siphonaria; specificEpithet: *japonica*; scientificNameAuthorship: (Donovan, 1824); **Location:** country: Korea; locality: Dokdo-ri, Ulleung-eup, Ulleung-gun, Gyeongsangbuk-do; verbatimCoordinates: 37°14'20.12"N, 131°52'05.74"E; **Event:** eventDate: 2021-06-02
- **Type status:** Other material. **Occurrence:** individualCount: 1; occurrenceID: FB198DA4-1645-5901-BDFA-506AE0CDD712; **Taxon:** scientificName: *Siphonariajaponica*; kingdom: Animalia; phylum: Mollusca; class: Gastropoda; order: Siphonariida; family: Siphonariidae; genus: Siphonaria; specificEpithet: *japonica*; scientificNameAuthorship: (Donovan, 1824); **Location:** country: Korea; locality: Guryongpo-eup, Nam-gu, Pohang-si, Gyeongsangbuk-do; verbatimCoordinates: 35°57'11.5"N, 129°32'50.4"E; **Event:** eventDate: 2023-07-12
- **Type status:** Other material. **Occurrence:** individualCount: 1; occurrenceID: 1B68A9AE-75A2-505F-8F9E-E9AE8E3C17F9; **Taxon:** scientificName: *Siphonariajaponica*; kingdom: Animalia; phylum: Mollusca; class: Gastropoda; order: Siphonariida; family: Siphonariidae; genus: Siphonaria; specificEpithet: *japonica*; scientificNameAuthorship: (Donovan, 1824); **Location:** country: Korea; locality: Daejin-ri, Hyeonnae-myeon, Goseong-gun, Gangwon-do; verbatimCoordinates: 38°29'57.1"N, 128°25'38.7"E; **Event:** eventDate: 2024-03-20
- **Type status:** Other material. **Occurrence:** individualCount: 3; occurrenceID: C26236C2-BD2D-5ED5-94A0-A09287579017; **Taxon:** scientificName: *Siphonariajaponica*; kingdom: Animalia; phylum: Mollusca; class: Gastropoda; order: Siphonariida; family: Siphonariidae; genus: Siphonaria; specificEpithet: *japonica*; scientificNameAuthorship: (Donovan, 1824); **Location:** country: Korea; locality: Onpyeong-ro, Seongsan-eup, Seogwipo-si, Jeju-do; verbatimCoordinates: 33°24'03.7"N, 126°54'17.7"E; **Event:** eventDate: 2024-07-28
- **Type status:** Other material. **Occurrence:** individualCount: 2; occurrenceID: DE709C43-9BAD-5511-BB20-64DF8FE5144F; **Taxon:** scientificName: *Siphonariajaponica*; kingdom: Animalia; phylum: Mollusca; class: Gastropoda; order: Siphonariida; family: Siphonariidae; genus: Siphonaria; specificEpithet: *japonica*; scientificNameAuthorship: (Donovan, 1824); **Location:** country: Korea; locality: Sagye-ro, Andeok-myeon, Seogwipo-si, Jeju-do; verbatimCoordinates: 33°13'13.2"N 126°17'42.0"E; **Event:** eventDate: 2022-04-21

#### Description

Measurements: SL 10.5–15.45 mm, SW 7.5–12.24 mm, SH 2.81–4.7 mm.

Shell (Fig. 2c) thin, oval, small size (in examined specimens, SL 10.5–15.45 mm), medium-high in height (2/7 of its length). Exterior colour greyish-brown. Shell surface with 17–23 thin, yellowish white-coloured primary radial ribs. Interspaces between radial ribs sometimes with 1–2 secondary ribs. Apex eroded, located centrally in posterior one-third of shell. Shell margin weakly undulated. Anterior slope slightly convex, posterior slope shorter, nearly straight. Siphonal groove weakly prominent with two radial ribs on outer shell surface. Interior colour reddish-brown.

Radula (Fig. 3c, d) dentition formulae 29:1:29 (in specimens measuring SL 10.54 mm, SW 7.47 mm, SH 2.81 mm). Each transverse row with narrow central rachidian tooth flanked by symmetrical half rows with lateral teeth, decreasing in size outwards. Rachidian tooth (Fig. 3c) short, -approximately one-third length of lateral tooth, with sharply pointed cusp. Innermost lateral teeth (first 17 lateral teeth), each tooth consisting of mesocone and ectocone; mesocone with bicuspid or unicuspid (Fig. 3c); ectocone shorter, pointed. Outermost lateral teeth (remaining 12 lateral teeth) consisting of endocone, ectocone and mesocone; mesocone paddle-shaped, not angled; endocone and ectocone short, sharply pointed (Fig. 3d).

#### Distribution

Korea, China, Japan and Taiwan.

**Type locality**: Japan.

**Habitat**: On rocky substrate in low to high intertidal zones (Fig. 2cE).

#### Taxon discussion

Jenkins and Köhler (2024) designated a neotype for *S.japonica* due to the absence of the original type specimens and provided a detailed description of the shell morphology, reproductive system and morphological features of spermatophore. The shell morphology of the Korean *S.japonica* specimens match es well both by Donovan (1824)'s original description and the observations by Jenkins and Köhler (2024). Nevertheless, moderate variations in the number of ribs were observed in this study, with the number of primary ribs ranging from 17 to 23 and secondary ribs ranging from 1 to 2. These ranges differ from those reported by Jenkins and Köhler (2024), which included up to 20 primary ribs and 2 to 3 secondary ribs. In addition, Jenkins and Köhler (2024) considered the shell images of Figs. 1 and 4 of *S.japonica* described by Yoo (1976) to likely represent misidentification, without providing detailed information on their interpretation. Upon re-examination of the Figs. 1 and 4, we observed that the specimens show characteristics consistent with *S.japonica*, including 20–25 primary ribs with secondary rib interstices (though the exact number is ambiguous) and two primary ribs on the siphonal groove. Based on our integrated analyses of the morphology (including the number of radial ribs on the shell surface) and molecular sequence data, we have concluded that all the shell images in Yoo (1976), including Figs. 1 and 4, represent within the normal range of shell variations typically observed in *S.japonica*. In radula morphology, Hubendick (1946) provided a brief sketch of radula characters, based on only two specimens in his description of *S.japonica*. He provided a radula formula ranging from 32:1:32 to 40:1:40 and noted the presence of an ectocone on the innermost lateral teeth. Our examination of the Korean specimens revealed a radula formula of 29:1:29, which differs from those reported in Hubendick (1946), while the presence of an ectocone on the innermost lateral teeth was consistent with his findings. These observations suggest that the radula formula can vary within the same species.

*S.japonica* is commonly found in intertidal zones along the Korean coast, often occurring alongside *S.acmaeoides* and *S.sirius* (Fig. 2aA, Fig. 2dG). Their wide range of distribution and variations in shell morphology can lead to confusion in identification, resulting in past misidentifications in Korean illustrations as *S.sirius* (Yoo 1976) and *S.acmaeoides* (Kwon et al. 1993, Kwon 2001, Min et al. 2004). However, this species can be distinguished from other sympatric *Siphonaria* species by its thinner, regularly spaced primary ribs, siphonal groove with two radial ribs, undulated margin and dark brown-coloured interior (Fig. 2cF). Yokogawa et al. (2010) described two types from *S.japonica*, based on the combination of morphological, molecula, and ecological data, as the “tall form” and the “short form”. The tall form is characterised by weak radial ribs over the shell margin and alternating sized (thick and thin) radial ribs, while the short form has projected radial ribs over the shell margin and generally thick radial ribs. The Korean *S.japonica* morphologically matches the “tall form”. A comprehensive analysis of shell morphological data, coupled with molecular sequence information from broader sampling of this species, is required to confirm whether the “short form” of *S.japonica* is also distributed along the Korean sea coast, as previously reported in Japan (Yokogawa et al. 2010). Our phylogenetic tree indicates that *S.japonica* is positioned basal amongst selected Siphonariidae species in the north-western Pacific, with 99% bootstrap support value (Fig. 4). This species exhibits relatively high sequence divergence from other *Siphonaria* species, ranging from 28.89% (*S.zelandica*) to 33.81% (*S.subatra*) (Table 2).

### Siphonaria javanica

(Lamarck, 1819)

E3469275-ED77-5298-8B46-35E721D8F2A5

https://www.gbif.org/species/5859486

https://www.marinespecies.org/aphia.php?p=taxdetails&id=740945

Siphonaria (Siphonaria) javanica : Lee and Min 2002: 146 [checklist]; Min et al. 2004: 334, fig. 1054.
Siphonaria javanica : Noseworthy et al. 2007: 90 [checklist].

#### Distribution

Japan (Okinawa island), Malaysia, Palau, Indonesia and northeast Australia.

**Type locality**: Indonesia; Java Island.

#### Taxon discussion

This species was initially included by Lee and Min (2002) in their checklist. Later, Min et al. (2004) described the shell characters of this species as having a large shell size (SL 20 mm, SW 18 mm, SH 9 mm) and white primary ribs with 2–3 thin secondary ribs in the interspaces. However, the shell characters in their description do not align with the original description of *S.javanica* provided by Lamarck (1819) or the subsequent studies by Morrison (1972) and Jenkins and Köhler (2024). The original description of *S.javanica* is brief, noting that the species possesses ‘white radiating ribs and crenated margin’ (translation from Latin). Morrison (1972) further noted that *S.javanica* was readily distinguished from other congeneric species by its high conical shell with buttress-like strong white radial ribs. Jenkins and Köhler (2024) provided a further detailed description of *S.javanica* by designating a lectotype and refined its taxonomy by clarifying its diagnostic characteristics. Their study also emphasised that *S.javanica* possesses well-developed primary ribs that extend beyond the shell margin, resulting in a coarsely undulated shell margin. This contrasts with the shell image provided by Min et al. (2004), which depicts the species with a smooth shell margin. Given the restricted distribution range of *S.javanica* to Indonesia and Timor-Leste, as clarified by the recent revision in Jenkins and Köhler (2024), Morrison (1972) and the discordance between the shell description of Korean “*S.javanica*” samples and both the original and subsequent descriptions, it is likely that the previously reported Korean specimens of “*S.javanica*” are likely misidentifications of *S.japonica*. Further studies with broader taxon sampling are necessary to resolve this taxonomic issue.

### Siphonaria laciniosa

(Linnaeus, 1758)

AEDFA66A-7042-515A-B14C-85DC15B6D68E

https://www.gbif.org/species/5189835

https://www.marinespecies.org/aphia.php?p=taxdetails&id=215290

Siphonaria laciniosa : Kwon et al. 1993: 335, fig. 61-4.
Siphonaria (Siphonaria) laciniosa : Kwon 2001: 191, fig. 721.
Siphonaria (Mestosiphon) laciniosa : *Lee and Min 2002* [checklist]: 146, fig. 268; Min et al. 2004: 336, fig. 1059; Noseworthy et al. 2007: 90 [checklist].

#### Distribution

Persian Gulf, Red Sea, Samaoan Islands, Tonga, Fiji (Morrison 1972).

**Type locality**: India.

#### Taxon discussion

Kwon et al. (1993) first reported the occurrence of this species in Korea, noting its high shell height (SL 20 mm, SW 18 mm, SH 9.4 mm), with approximately 50 radial ribs and a pointed apex. Subsequent literature described this species as having less pronounced radial ribs, with white spots along the inner shell margin (Kwon 2001, Min et al. 2004), which are shell features also commonly observed in some individuals of *S.japonica*. Jenkins and Köhler (2024) recently designated *S.laciniosa* as a “*nomen dubium*”, highlighting the “utter confusion” in its original description and subsequent literature. Jenkins and Köhler (2024) noted that many type specimens attributed to *S.laciniosa* are either lost or represent mixed lots. Given the substantial taxonomic confusion regarding the validity of *S.laciniosa* and the morphological overlap between Korean specimens previously identified as *S.laciniosa* (Kwon et al. 1993, Kwon 2001, Min et al. 2004) and *S.japonica* (as observed in this study), it is likely that the earlier records of *S.laciniosa* in Korea represent misidentifications of *S.japonica*. As a result, we excluded *S.laciniosa* from the list of Korean *Siphonaria* species.

### Siphonaria rucuana

Pilsbry, 1904

8BE22268-594F-5877-8077-B1E578A98041

https://www.gbif.org/species/6126792

https://www.marinespecies.org/aphia.php?p=taxdetails&id=599440

Siphonaria rucuana : Kwon et al. 1993: 335, fig. 61-5.
Siphonaria (Siphonaria) rucuana : Kwon 2001: 191, fig. 722; Lee and Min 2002: 146 [checklist]; Min et al. 2004: 335, fig. 1055; Noseworthy et al. 2007: 90 [checklist].

#### Distribution

Japan (Okinawa Island).

**Type locality**: Japan; Ryukyu island (Okinawa Island).

#### Taxon discussion

Kwon et al. (1993) first reported this species in Korea, based on shell morphology, describing it as having a flatly convex shell with a brown apex curving posteriorly and brown intervals with secondary ribs. However, in contrast to this description, Pilsbry (1904) described *S.rucuana* as having a steeply conic shell with a glossy brown-coloured apex curving posteriorly and dusky-coloured intervals without secondary ribs. These characteristics are consistent with the recent revision of Jenkins and Köhler (2024). Yokogawa et al. (2010) divided *S.japonica* into two shell forms—“tall form” and “short form” — based on an integrative analysis of their morphology, habitat ecology and allelic frequencies. Compared to these two shell forms of *S.japonica*, the specimens previously described as *S.rucuana* in Korean waters (Kwon et al. 1993, Kwon 2001, Min et al. 2004) do not accord with the Pilsbry (1904)'s original description and morphological characters described in the recent revisional study (Jenkins and Köhler 2024). A re-evaluation of prior records of *S.rucuana* in Korea suggests that the specimens described in Kwon et al. (1993) correspond to the “tall form” of *S.japonica*, representing the original *S.japonica*. On the other hand, the other two reports (Kwon 2001, Min et al. 2004) are assumed to correspond to the “short form” of *S.japonica* discovered first by Yokogawa et al. (2010) in Japan. In addition to these morphological aspects, *S.rucuana* is known to have a limited geographic distribution, being reported exclusively from Okinawa Island, a subtropical region of Japan (Pilsbry 1904, Oyama et al. 1954, Okutani 2000, Okutani 2017). Given this restricted geographic distribution range and the morphological discrepancies observed in the Korean nominal records of “*S.rucuana*”, it is likely that these previous records in the Korean malacofauna result from misidentifications.

### Siphonaria sirius

Pilsbry, 1894

4C5BDB3B-8EA1-5E6A-AB09-F6176B84E416

https://www.gbif.org/species/6126791

https://www.marinespecies.org/aphia.php?p=taxdetails&id=740940

*Siphonariasirius Pilsbry 1894*: 9, 10; Pilsbry 1895: 5, 6, pl. 6, figs. 23–28; Hirase 1941: 94, pl. 121, fig. 16; Hubendick 1945: 29 (cited from Jenkins and Köhler (2024)); Kuroda and Habe 1952: 86 (cited from Jenkins and Köhler (2024)); Azuma 1960: 62 (cited from Jenkins and Köhler (2024)); Kuroda 1960: 43 (cited from Jenkins and Köhler (2024)); Baker 1964: 159 (cited from Jenkins and Köhler (2024)); Shikama 1964: 6 (cited from Jenkins and Köhler (2024)); Habe and Igarashi 1967: 28 (cited from Jenkins and Köhler (2024)); Inaba 1983: 145 (cited from Jenkins and Köhler (2024)); Trew 1983: 7 (cited from Jenkins and Köhler (2024)); Je 1989: 29 [checklist]; Morton and Morton 1983: 298, pl. 1K (cited from Jenkins and Köhler (2024)); Hylleberg and Kilburn 2003, 2003: 133; White and Dayrat 2012: 68 [checklist]; Dayrat et al. 2014: 269, fig. 5H; Jenkins and Köhler 2024: 115–118, figs. 43E, F, O, P, 44D, E.
Siphonaria (Siphonaria) sirius : Hubendick 1946: 50, 51, pl. 3, figs. 24–27.
Siphonaria (Mestosiphon) sirius : Habe and Kikuchi 1960: 60 (cited from Jenkins and Köhler (2024)); Kira 1962: 200, 201, text-fig, pl. 69, fig. 12a, b (cited from Jenkins and Köhler (2024)); Habe 1971: 15, pl. 4, fig. 12 (cited from Jenkins and Köhler (2024)).
Anthosiphonaria sirius : Kuroda et al. 1971: 483, 484, fig. 9; Inaba 1983: 145 (cited from) ; Je 1989: 29 [checklist]; Choe 1992: 748, 749, pl. 126, fig. 223; Fukuda et al. 1992: 76, pl. 23, fig. 361a, b (cited from Jenkins and Köhler (2024)).
Siphonaria laciniosa forma sirius : Christiaens 1980a: 79.
Siphonaria laciniosa : Springsteen and Leobrera 1986: 285, pl. 81, fig. 19 (non *Siphonarialaciniosa* (Linneaus, 1758); cited from Jenkins and Köhler (2024)).
Siphonaria subatra : Je 1989: 29 [checklist].
Siphonacmea oblongata : Je 1989: 29 [checklist].
Siphonaria (Anthosiphonaria) sirius : Kwon et al. 1993: 334, fig. 61-2; Higo et al. 1999: 402, G4977 [checklist]; Kwon 2001: 191, fig. 725; Min et al. 2004: 336, fig. 1058; Okutani 2000: 815, pl. 405, fig. 5; Noseworthy et al. 2007: 90 [checklist]; Okutani 2017: 1101, pl. 403. fig. 5.

#### Materials

- **Type status:** Other material. **Occurrence:** individualCount: 1; occurrenceID: D54B007E-530E-5A64-9E37-238C909DFC8B; **Taxon:** scientificName: *Siphonariasirius*; kingdom: Animalia; phylum: Mollusca; class: Gastropoda; order: Siphonariida; family: Siphonariidae; genus: Siphonaria; specificEpithet: *sirius*; scientificNameAuthorship: Pilsbry, 1894; **Location:** country: Korea; locality: Dokdo-ri, Ulleung-eup, Ulleung-gun, Gyeongsangbuk-do; verbatimCoordinates: 37°14'20.12"N 131°52'05.74"E; **Event:** eventDate: 2016-06-22
- **Type status:** Other material. **Occurrence:** individualCount: 1; occurrenceID: C0A81431-17F4-52C1-891F-62FF72C4302D; **Taxon:** scientificName: *Siphonariasirius*; kingdom: Animalia; phylum: Mollusca; class: Gastropoda; order: Siphonariida; family: Siphonariidae; genus: Siphonaria; specificEpithet: *sirius*; scientificNameAuthorship: Pilsbry, 1894; **Location:** country: Korea; locality: Sagye-ro, Andeok-myeon,Seogwipo-si, Jeju-do; verbatimCoordinates: 33°13'58.7"N 126°22'23.8"E; verbatimCoordinateSystem: 33°13'13.2"N 126°17'42.0"E; **Event:** eventDate: 2022-04-21
- **Type status:** Other material. **Occurrence:** individualCount: 2; occurrenceID: BEE8EC41-E8D8-5759-BB91-328F2C2C0A3E; **Taxon:** scientificName: *Siphonariasirius*; kingdom: Animalia; phylum: Mollusca; class: Gastropoda; order: Siphonariida; family: Siphonariidae; genus: Siphonaria; specificEpithet: *sirius*; scientificNameAuthorship: Pilsbry, 1894; **Location:** country: Korea; locality: Irun-myeon,, Geoje-si, Gyeongsangnam-do; verbatimCoordinates: 34°58'34.9"N 128°41'18.18"E; **Event:** eventDate: 2023-04-19
- **Type status:** Other material. **Occurrence:** individualCount: 3; occurrenceID: 2213E690-E902-5F33-99A8-212A4C10304D; **Taxon:** scientificName: *Siphonariasirius*; kingdom: Animalia; phylum: Mollusca; class: Gastropoda; order: Siphonariida; family: Siphonariidae; genus: Siphonaria; specificEpithet: *sirius*; scientificNameAuthorship: Pilsbry, 1894; **Location:** country: Korea; locality: Namyang-ri, Seo-myeon, Ulleung-gun, Gyeongsangbuk-do; verbatimCoordinates: 37°27'35.6"N 130°51'27.0"E; **Event:** eventDate: 2023-05-23
- **Type status:** Other material. **Occurrence:** individualCount: 2; occurrenceID: 37D006D8-B765-5917-A845-CD6DD8473CD9; **Taxon:** scientificName: *Siphonariasirius*; kingdom: Animalia; phylum: Mollusca; class: Gastropoda; order: Siphonariida; family: Siphonariidae; genus: Siphonaria; specificEpithet: *sirius*; scientificNameAuthorship: Pilsbry, 1894; **Location:** country: Korea; locality: Mendehaean-gil, Tongyeong-si, Gyeongsangnam-do; verbatimCoordinates: 34°50'37.8"N 128°26'36.0"E; **Event:** eventDate: 2023-06-21
- **Type status:** Other material. **Occurrence:** individualCount: 1; occurrenceID: 2A53BAB4-3A3B-59CA-8E8A-716A5BC80FD6; **Taxon:** scientificName: *Siphonariasirius*; kingdom: Animalia; phylum: Mollusca; class: Gastropoda; order: Siphonariida; family: Siphonariidae; genus: Siphonaria; specificEpithet: *sirius*; scientificNameAuthorship: Pilsbry, 1894; **Location:** country: Korea; locality: Guryongpo-eup, Nam-gu, Pohang-si, Gyeongsangbuk-do; verbatimCoordinates: 35°57'05.7"N 129°33'04.4"E; **Event:** eventDate: 2023-07-23

#### Description

Measurements: SL 11.23–19.63 mm, SW 9.09–15.62 mm, SH 2.87–4.93 mm.

Shell (Fig. 2d) solid, oval, small to medium in size (in examined specimens, SL 11.23–19.63 mm), low in height (about 1/4 of its length). Exterior colour greyish-brown or blackish-brown. Shell surface with 5­–11 strong, white-coloured primary radial ribs. Interspaces between the primary radial ribs with 1­–8 thinner secondary ribs, coloured white, sometimes same as shell colour. Apex often eroded, located central of the shell. Shell margin prominently undulated by primary ribs. Anterior slope slightly convex, posterior slope nearly straight. Siphonal groove protruded with one radial rib on outer shell surface. Interior colour reddish-brown to black with white bands, sometimes white or pale-coloured at centre.

Radula (Fig. 3e, f) dentition formulae 34:1:34 (in specimens measuring SL 14.69 mm, SW 11.80 mm, SH 3.89 mm). Each transverse row with narrow central rachidian tooth flanked by symmetrical half rows with lateral teeth, decreasing in size outwards. Rachidian tooth (Fig. 3e) short, approximately one-third length of lateral tooth, bud-shaped, middle widest, tapering gently towards the top with sharply-pointed cusp. Innermost lateral teeth (first 15 lateral teeth), each tooth consisting of mesocone and ectocone; mesocone bicuspid, with U-shaped cleft; ectocone shorter, sharp (Fig. 3e). Outermost lateral teeth (remaining 19 lateral teeth) with short and sharply-pointed endocone, ectocone and mesocone; mesocone subquadrate (Fig. 3f).

#### Distribution

Korea, China, Japan, Philippines, Singapore, Indonesia (Sumatra Island) and Vietnam.

**Type locality**: Japan; Sagami, Kashiurazaki, Boshiu (Boso Peninsula).

**Habitat**: On rocky substrate in middle to low intertidal zones (Fig. 2dG).

#### Taxon discussion

This species is clearly distinguished from other Korean sympatric species (*S.acmaeoides* and *S.japonica*) by its flat shell, remarkably extended white radial ribs, siphonal groove with a single radial rib and pale-coloured inner centre (Fig. 2d). It closely resembles *S.atra* Quoy and Gaimard, 1833, by having a flat shell and strongly stretched radial ribs. Christiaens (1980a) noted that *S.sirius* represents one of the three forms of *S.laciniosa* (Linnaeus, 1758); (S.laciniosaformasirius, S.laciniosaformaatra (representing *S.atra* Quoy and Gaimard, 1833) and S.laciniosaformasubatra (representing *S.subatra* Pilsbry, 1904). He reported no differences in niche selection between *S.atra* and *S.sirius*, both of which inhabit highly exposed rocky substrates in the low tidal zone at Ping Chau, Hong Kong. However, the two species differ in colouration and the number of ribs in the siphonal groove. While *S.atra* has a dusky brown shell with radial ribs of the same colour (sometimes paler than the interspaces) and a siphonal groove with two radial ribs, *S.sirius* is characterised by a black or dark brown-coloured shell with solid white radial ribs and a siphonal groove with only one radial rib. In the radula formula, as mentioned in the cases of *S.acmaeoides* and *S.japonica*, *Siphonaria* species exhibits high levels of intraspecific variation. Similarly, the radula formulae of *S.sirius* reported in previous studies showed considerable variation depending on the authority, ranging from 40:1:40 (Hubendick 1946) to 34:1:34 (Christiaens 1980a). The radula formula (34:1:34) observed in this study is consistent with the finding of Christiaens (1980a). The phylogenetic tree shows that *S.sirius* forms a sister group with *S.atra* and *S.subatra* receiving 99% bootstrap value (Fig. 4). The *p*-distances within *S.sirius* species range from 0.16% to 0.79%, while this species shows high sequence divergence from other congeneric species ranging from 22.38% (*S.atra*) to 31.90% (*S.japonica*) (Table 2).

## Discussion

Previously, a total of seven *Siphonaria* species have been sparsely reported in Korea, based solely on external shell morphologies that has often resulted in taxonomic complications, including misidentification of species due to their remarkably high ecophenotypic variations. From a comprehensive analysis of shell morphology, radula structure and molecular analysis of mtDNA *cox1* sequences in this study, three species (*S.acmaeoides*, *S.japonica* and *S.sirius*) have been confirmed to occur in Korea. The three Korean *Siphonaria* species commonly share the presence of secondary ribs and often exhibit an eroded apex as they grow (Fig. 2). However, they are distinguished from each other by the number of primary ribs: 14–17 (in *S.acmaeoides*), 17–23 (in *S.japonica*) and 5–11 (in *S.sirius*), respectively. Furthermore, their primary ribs vary in thickness, elevation and spacing: *S.acmaeoides* has wide, low, unevenly spaced ribs; *S.japonica* has thin, moderately elevated, evenly spaced ribs; and *S.sirius* has wide, moderately elevated, unevenly spaced ribs. The morphology of the siphonal groove also varies amongst the species: *S.acmaeoides* has an indistinct groove with uncountable radial ribs, *S.japonica* has a weakly projected groove with two radial ribs and *S.sirius* has a strongly projected groove with a single radial rib. Very recently, Jenkins and Köhler (2024) compared the three species (*S.acmaeoides*, *S.japonica* and *S.sirius*) in their shell morphology and reproductive system, including the morphology of the spermatophore. Their shell morphology comparison focused on a set of characters such as the solidity and crenulation of the shell margin, which are less informative for distinguishing sympatric species due to substantial intraspecific variation that complicates precise species identification. Nevertheless, they provided anatomical comparisons, noting that *S.acmaeoides* differs from *S.japonica* by having a smaller accessory organ and bursa copulatrix, a shorter flagellum and a short drop-like spermatophore. In contrast, *S.sirius* has a larger accessory organ and a spermatophore lacking barbs. In radula morphology, *S.acmaeoides* has only a mesocone in the innermost lateral teeth, whereas both *S.japonica* and *S.sirius* possess a mesocone with sharply-pointed ectocone in the inner lateral teeth (Fig. 3). Despite these distinct morphological features, the radula formulae described in previous studies, including our observations, showed considerable individual variations (Hubendick 1946, Christiaens 1980a).

Our phylogenetic analysis using mtDNA *cox1* sequences of these three Korean *Siphonaria* species and some other congeneric species confirmed their morphology-based species identification (Fig. 4). The resulting tree indicated that the mtDNA *cox1* sequences determined from *S.acmaeoides*, *S.japonica* and *S.sirius* respectively formed monophyletic groups, corroborating their identification based on morphological characteristics. A significantly high sequence gap was observed between the lowest intraspecific distance (3.33%) and the highest interspecific distance (27.94%) amongst these three species. Interestingly, *S.acmaeoides* was found as closely related to *S.zelandica*, showing a relatively low genetic divergence (5.71–6.51%; Table 2). *S.acmaeoides* is known to be distributed in Japan, Korea (Kuroda et al. 1971, Choe 1992, Okutani 2000, Cheng et al. 2005, Yokogawa et al. 2010, Okutani 2017), whereas *S.zelandica* is found along the southern coasts of Australia (Quoy and Gaimard 1833, Jenkins 1983). Dayrat et al. (2014) proposed the possibility of an extensive distribution of *S.zelandica* ranging from Japan to Australia and suggested that *S.acmaeoides* could be a junior synonym of *S.zelandica*. However, Jenkins and Köhler (2024) confirmed that *S.acmaeoides* and *S.zelandica* are distinct species, based on comparative analyses of shell morphology, soft-body anatomy and mitochondrial phylogenetics. Unlike the three *Siphonaria* species confirmed to occur in Korea, the taxonomic validity of the other four species (*S.coreensis*, *S.javanica*, *S.laciniosa* and *S.rucuana*), previously reported from Korean waters is questionable. Despite extensive follow-up efforts over nearly the last two decades, these species have rarely been found. In particular, the shell images and morphological features described in the previous Korean literature do not closely match the original descriptions, but instead appear very similar to *S.japonica*, one of the most widely and abundantly found *Siphonaria* species in the north-western Pacific, including the Korean coastline. Moreover, none of these studies provided detailed descriptions of diagnostic characters and their species identifications were based solely on shell characteristics, raising questions about the taxonomic validity of their conclusions.

In conclusion, many molluscan species, including *Siphonaria* species, exhibit a high degree of morphological plasticity in shell shape (including sculptures), size and colour, which can vary significantly depending on environmental conditions. These variations in shell morphology make it challenging to differentiate species based on morphology alone, often resulting in various taxonomic errors, such as misidentifications, overlooking cryptic species diversity or a plethora of nominal species. Our taxonomic review incorporating both morphological and molecular data revealed that Korean *Siphonaria* species also display a wide range of shell morphological variations within species (e.g. shell colour, number of radial ribs and the slope of the anterior and posterior shell). Moreover, in many cases — including those observed in the present study — some species exhibiting a wide range of shell morphological variations are often found co-occurring in the same intertidal habitats. A comprehensive analysis of integrated morphological and molecular data, obtained through extensive taxon sampling along the Korean coastline can provide better resolution to address taxonomic ambiguities amongst *Siphonaria* species.

## Supplementary Material

XML Treatment for Siphonaria acmaeoides

XML Treatment for Siphonaria japonica

XML Treatment for Siphonaria javanica

XML Treatment for Siphonaria laciniosa

XML Treatment for Siphonaria rucuana

XML Treatment for Siphonaria sirius

## Figures and Tables

**Figure 2a.**
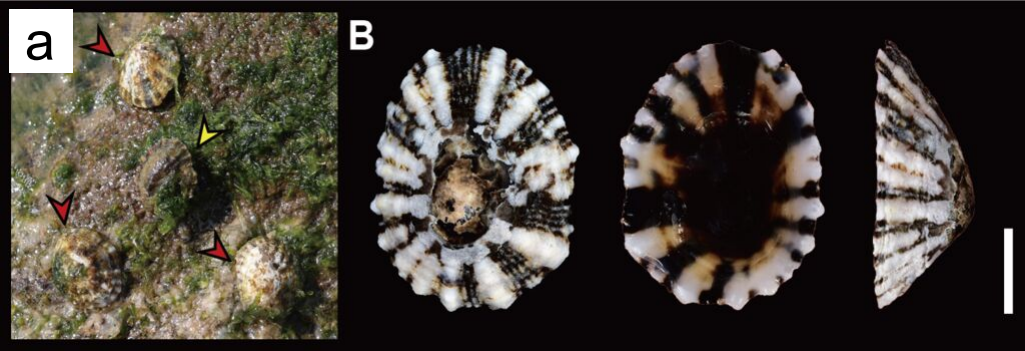
**A** *S.acmaeoides* on rocky substrate of Korea, Jeju; **B** Dorsal, ventral and lateral view (left) of *S.acmaeoides* from Jeju.

**Figure 2b.**
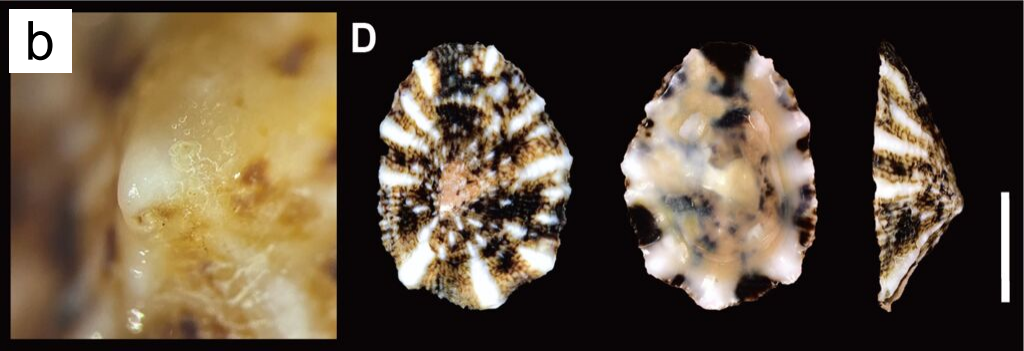
**C** Apex of *S.acmaeoides* juvenile from Jeju; **D** Dorsal, ventral, and lateral view (left) of *S.acmaeoides* juvenile from Jeju.

**Figure 2c.**
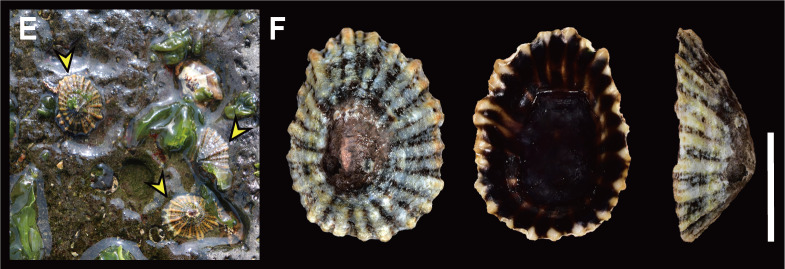
**E** *S.japonica* on rocky substrate of Korea, Jeju; **F** Dorsal, ventral and lateral view (left) of *S.japonica* from Goseong.

**Figure 2d.**
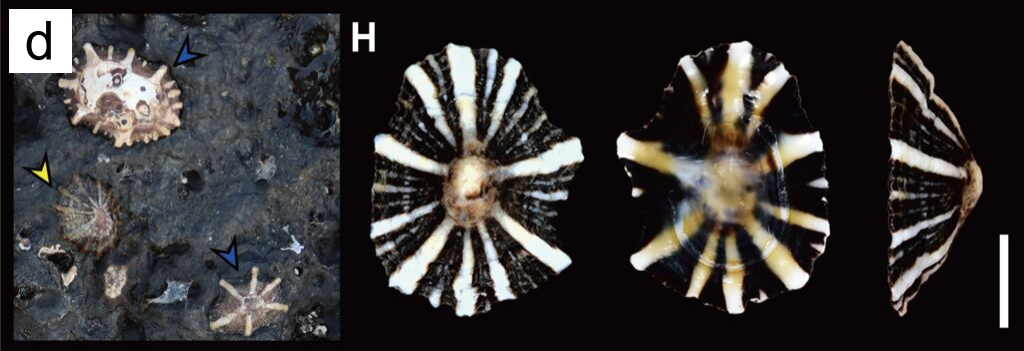
**G** *S.sirius* on rocky substrate of Korea, Jeju; **H** Dorsal, ventral and lateral view of *S.sirius* from Geoje.

**Figure 3a.**
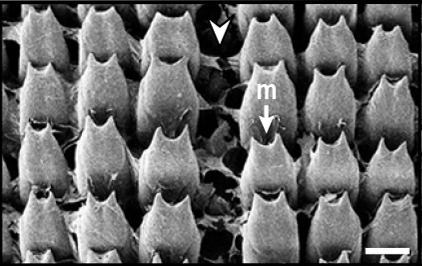
*S.acmaeoides* - Details of rachidian teeth (arrowhead) and innermost lateral teeth.

**Figure 3b.**
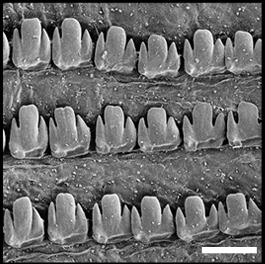
*S.acmaeoides* - Details of outermost lateral teeth.

**Figure 3c.**
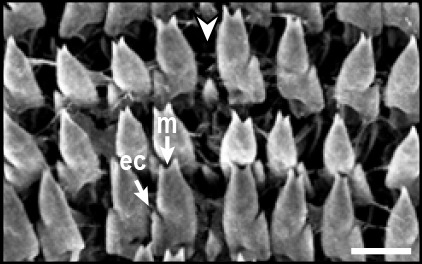
*S.japonica* - Details of rachidian teeth (arrowhead) and innermost lateral teeth.

**Figure 3d.**
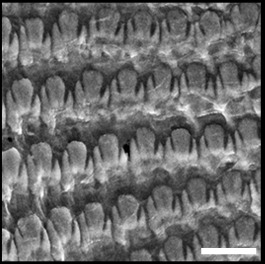
*S.japonica* - Details of outermost lateral teeth.

**Figure 3e.**
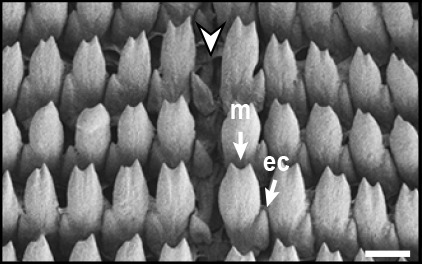
*S.sirius* - Details of rachidian teeth (arrowhead) and innermost lateral teeth.

**Figure 3f.**
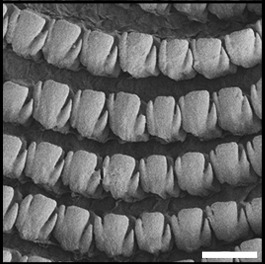
*S.sirius* - Details of outermost lateral teeth.

**Table 1.** Sampling localities and GenBank accession numbers of mtDNA *cox1* sequences of *Siphonaria* species used for molecular analysis.

**Species**	**Locality**	**GenBank accession nos.**	**References**
*S.acmaeoides*_01	Guryongpo-eup, Nam-gu, Pohang-si, Gyeongsangbuk-do	PQ422946	This study
*S.acmaeoides*_02	Sagye-ro, Andeok-myeon, Seogwipo-si, Jeju-do	PQ422947	This study
*S.acmaeoides*_03	Sagye-ro, Andeok-myeon, Seogwipo-si, Jeju-do	PQ422948	This study
*S.acmaeoides*_04	Hyeongjehaean-ro, Andeok-myeon, Seogwipo-si, Jeju-do	PQ422949	This study
*S.acmaeoides*_05	Sagye-ro, Andeok-myeon, Seogwipo-si, Jeju-do	PQ422950	This study
*S.atra*	China	OQ735434	Unpublished
*S.japonica*	Japan	KF000756	Dayrat et al. (2014)
*S.japonica*	Taiwan	KF000759	Dayrat et al. (2014)
*S.japonica*	Taiwan	KF000760	Dayrat et al. (2014)
*S.japonica*	Taiwan	KF000762	Dayrat et al. (2014)
*S.japonica*	Japan	KF000835	Dayrat et al. (2014)
*S.japonica*_01	Onpyeong-ro, Seongsan-eup, Seogwipo-si, Jeju-do	PQ422951	This study
*S.japonica*_02	Onpyeong-ro, Seongsan-eup, Seogwipo-si, Jeju-do	PQ422952	This study
*S.japonica*_03	Daejin-ri, Hyeonnae-myeon, Goseong-gun, Gangwon-do	PQ422953	This study
*S.javanica*	Singapore	KF690503	Ip et al. (2019)
*S.laciniosa*	Japan	KF000727	Dayrat et al. (2014)
*S.laciniosa*	Japan	KF000728	Dayrat et al. (2014)
*S.laciniosa*	Japan	KF000758	Dayrat et al. (2014)
*S.sirius*	Japan	KF000832	Dayrat et al. (2014)
*S.sirius*	Japan	KF000833	Dayrat et al. (2014)
*S.sirius*_01	Mendehaean-gil, Tongyeong-si, Gyeongsangnam-do	PQ422954	This study
*S.sirius*_02	Nambu-myeon, Geoje-si, Gyeongsangnam-do	PQ422955	This study
*S.sirius*_03	Mendehaean-gil, Tongyeong-si, Gyeongsangnam-do	PQ422956	This study
*S.subatra*	Japan	KF000743	Dayrat et al. (2014)
*S.subatra*	Japan	KF000754	Dayrat et al. (2014)
*S.subatra*	Japan	KF000755	Dayrat et al. (2014)
*S.subatra*	Japan	KF000757	Dayrat et al. (2014)
*S.zelandica*	Australia	JX680967	Colgan and Da Costa (2013)
*S.zelandica*	Australia	KF000725	Dayrat et al. (2014)
*S.zelandica*	Australia	KF000791	Dayrat et al. (2014)
*S.zelandica*	Australia	KF000793	Dayrat et al. (2014)
*T.afer*	-	EF489388	Klussmann-Kolb et al. (2008)
*T.reticulatus*	-	JN632509	White et al. (2011)

**Table 2.** Uncorrected *p*-distance (%) for the mtDNA *cox1* sequences amongst *Siphonaria* species. The species which include newly-determined species in this study are denoted by asterisks (*).

	S.japonica*	*S.zelandica*	S.acmaeoides*	*S.javanica*	*S.laciniosa*	S.sirius*	*S.atra*	*S.subatra*
S.japonica*	0.16–1.75							
*S.zelandica*	28.89–29.84	0.16–1.90						
S.acmaeoides*	29.37–30.95	5.71–6.51	0.16–3.33					
*S.javanica*	29.52–30.32	29.05–30.00	29.37–30.16	-				
*S.laciniosa*	29.05–30.14	28.57–29.52	28.57–29.68	15.56–16.03	0.16–0.79			
S.sirius*	30.79–31.90	28.25–29.68	27.94–29.21	30.63–30.79	29.52–30.48	0.16–0.79		
*S.atra*	31.27–31.90	27.78–28.25	28.25–28.82	27.46	27.14–27.30	22.38–22.86	-	
*S.subatra*	31.90–33.81	29.68–30.63	29.37–30.63	29.68–30.79	29.21–30.48	25.24–25.87	15.87–16.51	2.06–3.33
